# Editorial: Imaging technology in oncology pharmacological research, volume II

**DOI:** 10.3389/fphar.2022.977434

**Published:** 2022-08-08

**Authors:** Qi Zeng, Xu Cao, Jinchao Feng, Hong Shan, Xueli Chen

**Affiliations:** ^1^ School of Life Science and Technology, Xidian University & Engineering Research Center of Molecular and Neuro Imaging, Ministry of Education, Xi’an, China; ^2^ Xi’an Key Laboratory of Intelligent Sensing and Regulation of Trans-Scale Life Information & International Joint Research Center for Advanced Medical Imaging and Intelligent Diagnosis and Treatment, School of Life Science and Technology, Xidian University, Xi’an, China; ^3^ Innovation Center for Advanced Medical Imaging and Intelligent Medicine, Guangzhou Institute of Technology, Xidian University, Guangzhou, China; ^4^ Thayer School of Engineering, Dartmouth College, Hanover, IN, United States; ^5^ Faculty of Information Technology, Beijing University of Technology, Beijing, China; ^6^ Department of Interventional Medicine, Guangdong Provincial Engineering Research Center of Molecular Imaging, The Fifth Affiliated Hospital of Sun Yat-Sen University, Zhuhai, China

**Keywords:** imaging technology, pharmacological research, fluorescence imaging, PET/CT radiomics, oncology

Nowadays, imaging technology is well acknowledged as an important tool for drug development. Imaging could provide more detail and precise morphology or functionality information in gene expression, metabolism of various substances, cancer detection, drug development, as well as other fields. Similar to the previous Volume I, the research topic “Imaging Technology in Oncology Pharmacological Research, Volume II” provides an academic platform to discuss the latest oncology pharmacological works based on imaging technology.

Imaging technology could be a powerful tool both in the fast-screening of drugs *in vitro* and evaluating pharmacology *in vivo*. Liu et al. in their article, described a fluorescent probe G-Flamp2 with improved brightness and larger maximum ΔF/F_0_ directly derived from G-Flamp1 (their previous work, Liang et al., 2022). It could be applied for image-based high-content screening (HCS) to identify and evaluate the effect of candidate compounds targeting GPCR-cAMP signaling pathways. In an article by Yu et al., entitled, a marine-derived natural product terphenyllin was proved as a potential STAT3 inhibitor through *in vitro* and *in vivo* experiments based on high-through virtual structural-based screening. Terphenyllin was identified to exert inhibitory effects on the growth and metastasis of gastric cancer at an effective dose without significant toxicity by inhibiting the STAT3 signaling. Fluorescence images were applied to monitor the tumor metastasis by orthotopic implantation and evaluate *in vivo* efficacy of antitumor drugs.

Notably, imaging technology (especially PET/CT) combined with deep learning would provide more assistant information and accurate predictions for clinical anti-cancer medication strategies. In Huang et al., they established a hybrid model combining the smoking characteristic and deep learning features based on PET/CT images in diagnosing epidermal growth factor receptor (EGFR) mutation status of non–small cell lung cancer (NSCLC) patients. The proposed hybrid model achieved the best diagnostic performance in predicting EGFR mutation status for NSCLC patients based on PET/CT images of 194 patients, which could enable NSCLC patients to choose personalized treatment schemes. Feng et al. also focused on improving the prediction model of EGFR mutation status. In, they constructed an ensemble mode using l, 409 radiomics features extracted from CT images of 168 patients with NSCLC. They verified the predicting ability of the EGFR mutation status.And found this ensemble model could improve almost all indexes, especially reduce the false-positive rate significantly. Wang et al., in their research, employed knowledge of EGFR mutation status to improve the accuracy of the radiomics score models for predicting KRAS gene status based on ^18^F-FDG PET/CT multimodality imaging data. They proposed a composite model combining mixedRS and EGFR to analyze 258 NSCLC patients and found an improvement in prediction performance after integrating EGFR mutation status in radiomics models.

We believe that this topic shows the latest studies regarding applications of imaging technology in oncology pharmacological research. A collection of five research articles contributed to this research topic indicates that fluorescence imaging plays a useful role in drug screening and response evaluation both *in vitro* and *in vivo*. While, PET/CT radiomics is a powerful tool in clinical prediction, analysis and optimization for the personalized treatment. These articles in Volume II continue to embody the value of imaging technology in both basic research and clinical oncology pharmacological research like the previous Volume I.

## References

[B1] Liang C. W. Wang L. Peng W. L. Zhou Z. L. Zeng J. Z. Li X. L. (2022). A high-performance genetically encoded fluorescent indicator for *in vivo* cAMP imaging. BioRxiv (June 22, 2022). 10.1101/2022.02.27.482140 PMC9468011 36097007

